# The Crucial Role of Biofilms in *Cryptococcus neoformans* Survival within Macrophages and Colonization of the Central Nervous System

**DOI:** 10.3390/jof3010010

**Published:** 2017-02-24

**Authors:** Lilit Aslanyan, David A. Sanchez, Silvana Valdebenito, Eliseo A. Eugenin, Raddy L. Ramos, Luis R. Martinez

**Affiliations:** 1 Department of Biomedical Sciences, NYIT College of Osteopathic Medicine, New York Institute of Technology, Old Westbury, NY 11568-8000, USA; laslanya@nyit.edu (L.A.); rramos02@nyit.edu (R.L.R.); 2 Howard University College of Medicine, Washington, DC 20059-1027, USA; dsanch1988@gmail.com; 3 Public Health Research Institute and Department of Microbiology and Molecular Genetics, New Jersey Medical School, Rutgers University, Newark, NJ 07103-9998, USA; sv505@njms.rutgers.edu (S.V.); Eliseo.Eugenin@rutgers.edu (E.A.E.)

**Keywords:** biofilms, CNS, *Cryptococcus neoformans*, cryptococcomas, macrophages

## Abstract

*Cryptococcus neoformans* is an encapsulated yeast-like fungus capable of causing life threatening meningoencephalitis in patients with impaired immunity. This microbe primarily infects the host via inhalation but has the ability to disseminate to the central nervous system (CNS) either as a single cell or inside of macrophages. Upon traversing the blood brain barrier, *C. neoformans* has the capacity to form biofilm-like structures known as cryptococcomas. Hence, we will discuss the *C. neoformans* elements contributing to biofilm formation including the fungus’ ability to survive in the acidic environment of a macrophage phagosome and inside of the CNS. The purpose of this mini-review is to instill fresh interest in understanding the importance of biofilms on fungal pathogenesis.

## 1. Introduction

*Cryptococcus neoformans* is an encapsulated yeast-like fungus capable of infections ranging from asymptomatic colonization of lung tissue to life-threatening meningoencephalitis. There are approximately 1,000,000 yearly reported cases of cryptococcosis worldwide, of which ~625,000 lead to death within the first 3 months of infection [1]. Although commonly affecting individuals with AIDS, symptomatic cryptococcosis has also been observed in solid organ transplant recipients, cancer patients, intravenous drug users, and individuals with various autoimmune conditions requiring immunosuppressive drug therapy [2,3,4].

The primary infectious process begins by inhalation of aerosolized cryptococcal cells. As the pathogen navigates down the bronchial tree, it encounters alveolar macrophages which readily internalize the fungus. In many individuals, however, infection is not completely cleared and instead persists in a latent asymptomatic state that can progress to acute outbreaks in the event of host immunosuppression [5]. This form of virulence is achieved by the ability of *C. neoformans* to survive within the harsh acidic phagosomal environment, proliferate inside of macrophages despite nutrient deprivation, and disseminate to extra pulmonary sites [6,7]. By remaining hidden within macrophages and undetected by the host immune system, the cryptococci are able to disseminate and invade different organ systems, having particular affinity for the central nervous system (CNS). Furthermore, *C. neoformans* has developed the intricate strategy of phagosomal extrusion where the fungus can exit macrophages without damaging the host effector cells [8].

Because *C. neoformans* is found in association with soil-containing pigeon excreta [9] and has long been subject to radiation [10] as well as predation in this challenging environment [11,12], defensive mechanisms have evolved where the mode of growth and survival occur in specifically arranged communities structured for protection against polymicrobial competition [13,14]. Indeed, these microbial communities, called biofilms, are the most common form of growth in up to 80% of microorganisms in nature [15]. A major component of its polysaccharide capsule, glucuronoxylomannan (GXM), greatly contributes to the pathogenesis of cryptococcal disease [16]. In addition to disrupting host immune response mechanisms, GXM plays a central role in biofilm formation [17]. When compared to their free floating planktonic counterparts, biofilm-associated cryptococcal cells display a stable association with biological membranes that protect against host immune defenses [18] and antimicrobial therapy [19] in a self-produced polysaccharide rich exopolymeric matrix (EPM) [20]. Moreover, case reports of prosthetic heart valve endocarditis [21] and ventriculoatrial shunt [22] cryptococcal infections showcase the microbe’s biofilm forming capacity on medical devices and therefore stress the importance of investigating virulence factors associated with *C. neoformans* pathogenesis.

In this mini-review, we discuss the importance of biofilms in the pathogenesis of cryptococcal infection. We describe the importance of the polysaccharide capsule in biofilm formation and survival by *C. neoformans* within the host despite the continuous attack of the immune mechanisms. We evaluate the strategies in the context of biofilm function leading to *C. neoformans* survival inside macrophages and ability to cross biological membranes, particularly the blood brain barrier (BBB). Finally, we discuss *C. neoformans* survival in the CNS and the health complications of biofilm-like cryptococcomas. Our objective is to present the latest information on cryptococcal biofilms in the setting of infection and stimulate future research to enhance our current knowledge of the biology of *C. neoformans*.

### 1.1. C. neoformans Polysaccharide Capsule is Essential for Biofilm Formation and Pathogenesis

*C. neoformans* polysaccharide capsule is one of its critical virulence factors since the acapsular variants have shown reduced pathogenicity [23]. For instance, upon deletion of the gene CAP59, *C. neoformans* became capsule-deficient creating an avirulent phenotype [24]. Attenuated virulence in acapsular variants is compounded by the inability to form biofilms even when supplemented with exogenous GXM, suggesting that active production of the capsular polysaccharide is required [17]. These findings suggest that an ability to synthesize capsular components is essential for pathogenicity, including capsular growth and biofilm production. 

Copious amounts of capsular polysaccharide are shed during cryptococcal biofilm formation, creating an elaborated EPM or three-dimensional framework that encases the cell population for maximal protection and mechanical stability [17,20]. Chemical analysis of the EPM composition has shown the predominance of xylose, mannose, and glucose and the presence of several minor sugars not found in *C. neoformans* capsular polysaccharides [20]. Since GXM does not contain glucose, it is possible that the EPM is composed of types of polysaccharides that are different from those used to assemble the capsule. 

Self-aggregation of the polymers of the capsular polysaccharide is mediated by interactions between the carboxyl groups of glucuronic acid residues and divalent cations [25]. Evidently, calcium and magnesium ion concentrations have a direct effect on capsule assembly, where extracellularly accumulated GXM aggregates become incorporated into the apically growing yeast cell capsule [25,26]. The involvement of these ions in this process is supported by smaller observed capsular sizes in the presence of the divalent cation chelating agent EDTA [25]. In a similar manner, EDTA also inhibits biofilm formation, extracellular vesicle secretion, along with reduced amounts of GXM released by *C. neoformans*, emphasizing the requirement of divalent cations as well as GXM polymers in the formation of the EPM needed to facilitate, coordinate and stabilize the biofilm framework [27]. Notably, magnesium ions act as a signaling molecule, inducing capsule biosynthesis by promoting CAP gene expression [28]. Thus, divalent cations may be providing both mechanical and chemical support by participating in the production and aggregation of GXM polymers, an important initial step in cryptococcal biofilm formation.

To examine GXM production and secretion methods, *C. neoformans* was incubated with antibodies targeting surface epitopes. Microscopic data using specific antibodies indicated the presence of GXM inside the polysaccharide capsule, cell wall, and cytoplasm, suggesting that at least part of the secreted GXM polymers are intracellularly synthesized [29]. A proposed mechanism of secretion is through the packaging and export of vesicles, transporting GXM along with many other yeast components into the extracellular milieu [30]. Besides containing the main building blocks for capsular and biofilm growth, these “virulence bags” carry *C. neoformans* derived factors that can potentially compromise host cellular mechanisms [31]. This was validated by performing fractionation, structural imaging, and proteomic analyses of *C. neoformans* culture supernatants that revealed around 20–400 nm vesicles with variable morphology and density. These extracellularly secreted structures contained diverse proteins of which 76, including enzymes laccase and urease, were identified as vesicular components [31]. Furthermore, GXM-specific antibodies were shown to recognize *C. neoformans* extracellular vesicles, strongly corroborating the intracellular origin of GXM [32].

Antibodies targeting GXM have also lead to reduced secretion of this capsular component by *C. neoformans* [33]. The proposed mechanism of this outcome is that the antibodies cross link the GXM polymers likely leading to increased capsular rigidity and thus mechanically blocking the release of GXM, perhaps even GXM-containing vesicles [33,34]. In contrast, GXM-specific IgG_1_ 18B7 co-administered with antifungal agents showed decreased pharmacological efficacy against cryptococcal biofilms [35]. A conceivable explanation to the antagonism is that cross linking of GXM in the EPM prevents the relatively large molecules of the antifungal agents from accessing cryptococcal cells. Formation of biofilms is not only an effective method of protection from immune attacks but can also increase drug resistance by favorably modifying the intricate architecture with host antibodies during infection. 

### 1.2. C. neoformans Survival within Macrophages is Associated with Biofilm-Like Formation

Successful eradication of a cryptococcal infection requires a distinctive adaptive immune response profile. Th1 and Th17 cell cytokines, including IFN-γ, TNF-α and IL-17, are known inducers of anti-cryptococcal activity [36]. In contrast, Th2 cytokines IL-4 and IL-13 significantly increase intracellular yeast cell survival and proliferation [36]. This immune response elucidates why *C. neoformans* is extremely dangerous in HIV-infected patients, who with detectable cytokine shifts from Th1 to Th2 profile, lack effector antimicrobial molecules and retain ones that promote cryptococcal growth [37]. However, due to the long period required for adaptive immune response activation, the innate immune system will quickly shield the host from fungal cells with pulmonary dendritic cells and macrophages, which readily internalize the opsonized yeast. In fact, depletion of both cells types one day prior to infection caused severe disease in mice, consequently leading to death within 6 days after *C. neoformans* inoculation [38].

Avoidance of phagocytosis in order to escape from oxidative stress and nutrient deficiency is central to *C. neoformans* pathogenesis. One way of evading phagocytosis is accomplished by changing its cell morphology, transforming into a titan cell [39,40]. Due to the size of these giant yeast cells, macrophages are not able to ingest them, thus leading to extracellular environment colonization for easy access to tissue invasion [41]. Similarly, *C. neoformans* biofilm formation and water channel structures within biofilms protect densely-packed cryptococci from antimicrobial damage and macrophage phagocytosis in tissues enhancing fungal resistance, quorum sensing, and survival [17]. *C. neoformans* also expresses the anti-phagocytic protein 1 (App1), which specifically inhibits the CD11b domain of complement receptor 3 (CR3) to prevent phagocytosis of iC3b opsonized fungal cells [42]. Because antibody opsonized cells are internalized via the Fcγ receptor and not CR3, App1 does not inhibit antibody-mediated phagocytosis [42]. Additionally, the negatively charged *C. neoformans* capsule, due to its main component GXM, helps create electrostatic repulsive forces preventing cell-cell contact required for phagocytosis [43]. Despite possessing these anti-phagocytic abilities, *C. neoformans* is a facultative intracellular pathogen, able to survive inside of macrophages, particularly in the acidic phagosome [6,7].

In most primary infections, acidification of the phagosomes in immune cells is a necessary step in the process of eliminating internalized infectious agents. However, in the case of *C. neoformans*, this process in macrophages creates a rather favorable environment, where the pathogen is able to survive and proliferate under low pH conditions [7]. Moreover, immunofluorescence studies of late lysosomal marker LAMP-1 show proper phagolysosomal fusion in *C. neoformans* infected macrophages, suggesting this immune response is beneficial for the yeast [15]. In addition, it is possible that the low pH within the phagolysosome promote fungal modifications of the capsular molecules size and assembly for survival in this challenging milieu. The importance of acidic conditions for *C. neoformans* is highlighted with chloroquine treatment, where the weak base accumulates in yeast containing phagosomes, causing increases in pH and subsequently lowering the microbial proliferation rate [44]. This finding not only stresses the importance of acidic environments for fungal growth but also brings forth a model of cryptococcal survival in the CNS.

Although phagosomal conditions are ideal for *C. neoformans* survival, an eventual problem the pathogen will encounter in this limited space is nutrient starvation. However, the yeast can permeabilize phagolysosomal membranes, an action likely attributed to cryptococcal phospholipases, resulting in nutrient displacement into the compartment from host cell cytoplasm [45]. At the same time, there in an associated increase in the phagosomal pH as well as accumulation of *C. neoformans* produced polysaccharide rich vesicles throughout the cytosolic space of the macrophages. Consistent with bidirectional exchange of cellular materials, this direct host immune cell exposure to cryptococcal cells will allow the yeast to manipulate cell machinery and prevent microbial killing. Studies demonstrating that purified soluble cryptococcal GXM induces FasL expression on macrophages ultimately leading to apoptosis of activated T-cells expressing Fas supports this notion [46]. This kind of cytotoxicity of host immune cells therefore decreases adaptive immune responses against *C. neoformans*.

**Figure 1:**
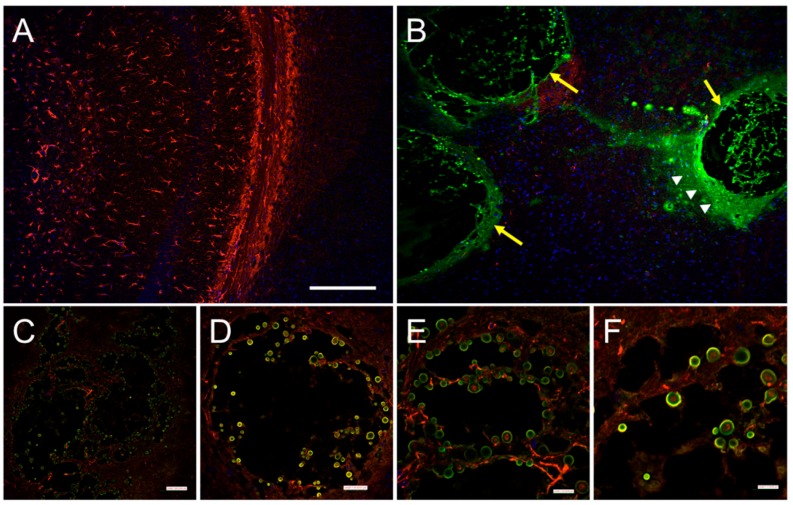
Biofilm-like cryptococcomas (brain lesions) in hippocampal tissue sections 14 days after intratracheal inoculation of *C. neoformans* strain H99 in a C57BL/6J mouse. (**A**) Confocal microscopy of the hippocampus in the brain of an infected animal with *cap59* (acapsular mutant). Capsule deficient mutants are cleared by phagocytic cells in the lungs of infected animals and are unable to reach the central nervous system; (**B**) Immunofluorescent image of a hippocampal tissue section of a mouse infected with wild-type *C. neoformans* H99 displaying large cryptococcomas (yellow arrows) filled with yeasts cells and abundant amounts of capsular polysaccharide released (white arrow heads) in the area; (**C**,**D**) Cryptococcomas are characterized by significant neuronal loss due to biofilm-like colonization of brain tissue; (**E**,**F**) High magnification images show a substantial number of yeast cells attached to neuronal tissue. For panels **A**–**F**, capsular-specific monoclonal antibody 18B7 (monoclonal antibody 18B7; green) was used to label fungal cells and capsular polysaccharide released. GFAP (red) and DAPI (blue) staining were used to label the cell bodies and nuclei of astrocytes, respectively. Scale bars: A,B = 230 µm; **C** = 87 µm; **D** = 35 µm; **E** = 21 µm; **F** = 17 µm.

Phagosomal permeabilization has also been observed prior to *C. neoformans* expulsion from macrophages [47]. Also known as phagosomal extrusion, this process serves as a safe exit strategy from inhospitable macrophages without any significant damage to the yeast or the host cell [8,46]. One pathogenic consequence of this escape mechanism is undetected dissemination to the CNS [46]. Shotgun proteomic data underscore that cryptococcal biofilm cells show increased expression of enzymes involved in proteolysis and protection from oxidative stress [48]. Interestingly, the mode of opsonization of *C. neoformans* prior to phagocytosis results in different morphologies of the emerging group of cells following expulsion. While complement-mediated phagocytosis results in individually dispersed cryptococcal cells, antibody-mediated processes lead to agglutination, and cryptococcal cells emerge in biofilm-like microcolonies [49]. Since microcolonies are a major structural subunit of biofilms, this form of dispersal allows *C. neoformans* to colonize new areas where the pathogen can potentially form biofilms in tissue, remain latent, and difficult to eradicate by macrophages, antimicrobial drugs, or molecules produced by immune cells. This is especially evident in brain tissue of patients with cryptococcosis where the fungus forms cryptococcomas or biofilm-like structures characterized by yeast cells surrounded by extensive amounts of capsular polysaccharide (Figure 1). In fact, the formation of cryptococcomas in the CNS by *C. neoformans* provides a plausible explanation for its successful neurotropism.

### 1.3. C. neoformans Biofilm Formation is Important for the Host CNS Invasion and Colonization

Infiltration of the CNS by *C. neoformans* can lead to meningoencephalitis, resulting in death, especially with the presence of underlying immunodeficiencies [50,51]. Therefore, it is important to investigate *C. neoformans* factors contributing to mechanisms that disrupt the integrity of the BBB. *C. neoformans* crosses the BBB transcellularly [52], across endothelial cells, paracellularly [53], between endothelial cells, or using the “Trojan horse mechanism” [54], undetected inside of peripheral macrophages. Dissemination to the CNS is likely accompanied by severe hypoxic stress due to the environmental shift from well aerated lungs. Indeed, it has been demonstrated that after crossing the BBB, cryptococcal cells colonize areas close to capillary endothelial cells to likely access oxygen rich blood [52]. Morphological changes and cystic biofilm-like adaptation following invasion of brain parenchyma may be directly involved with lower oxygen levels [52,55].

Biofilm formation by *C. neoformans* is an extremely complex and intricate process, requiring not only the presence of virulence factors but also favorable environmental conditions. Factors such as surface support and conditioning, fluid hydrodynamics, and microbial cellular properties interactively contribute to biofilm development [20]. For example, artificial cerebrospinal fluid (CSF) conditioned surfaces, along with favorable temperature and neutral pH levels are factors contributing to stronger biofilm formation [20]. CSF derived elements such as sugars, cationic compounds, and fluid hydrodynamics promote *C. neoformans* adhesion to medical devices and increase the stability of the biofilms [30]. For instance, colonization of a particular area is increased with surface roughness, a feature contributing to increased surface area, this consequently provides a broader interface for pathogen adhesion. 

Mannitol has been detected in the CSF of patients with cryptococcal meningitis [56]. A postulated function of mannitol includes facilitation of increased intracranial pressure due to a rise in CSF osmolality, resulting in severe neurologic damage in infected patients [57]. Moreover, certain cryptococcal transcription factors are involved in cell cycle regulation, which when activated can lead to G2 arrest under hypoxic conditions permitting *C. neoformans* survival under limited oxygen supply [55]. Expectedly, targeted deletions of genes involved in this process resulted in increased susceptibility to cell wall damage as well as reduced *C. neoformans* biofilm forming capacity [58]. Another change observed in cryptococcal biofilm cells is decreased levels of proteins involved in metabolic processes, suggesting a switch in energy production pathway from tricarboxylic acid or Krebs cycle to fermentation [48].

**Figure 2:**
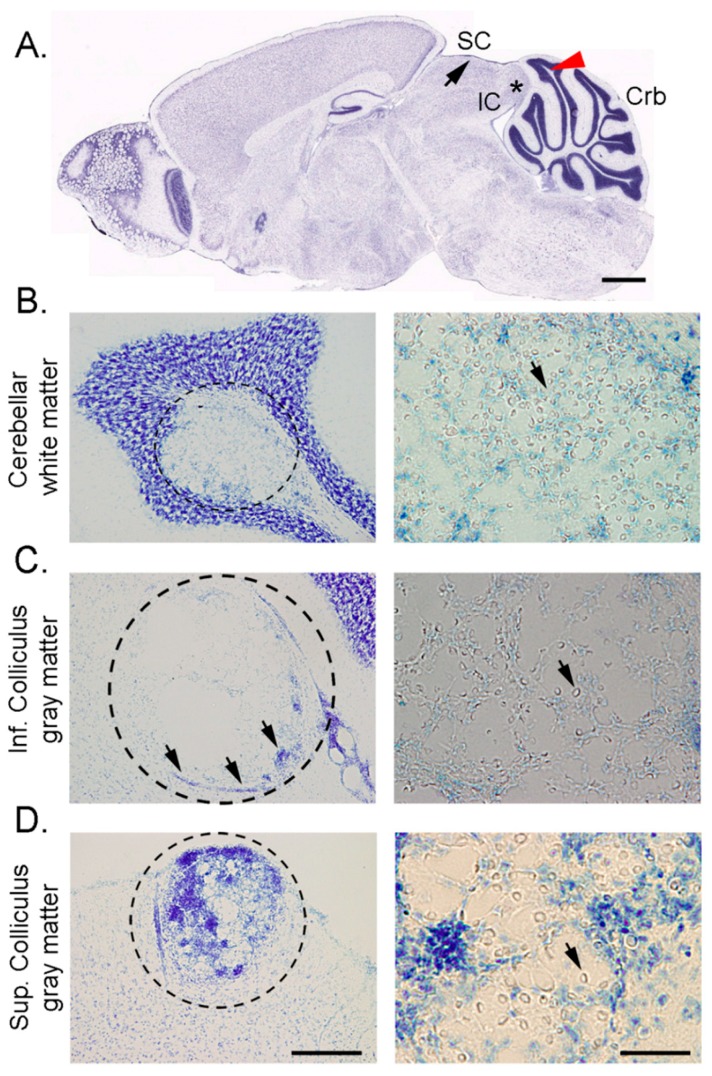
Cryptococoma formation in the gray and white matter of the midbrain and hindbrain 14 days after intratracheal inoculation of *C. neoformans* strain H99 in a C57BL/6J mouse. (**A**) Nissl-stained sagittal section indicating location of cyptococomas observed in the superior colliculus (SC; black arrow), inferior colliculus (IC; black asterisk), and cerebellum (Crb; red arrowhead); (**B**) Low- and high-magnification of a white matter cryptococoma (left and right panels) in the anterior cerebellum; (**C**) Low- and high-magnification of a white matter cryptococoma (left and right panels) in the inferior colliculus showing loss of parenchymal neurons and few macrophages except along the borders of the lesion (arrows); (**D**) Low- and high-magnification of a white matter cryptococoma (left and right panels) in the dorsal superior colliculus with marked presence of Nissl-stained microglia/macrophages. Panel A was adapted from the Allen Brain Mouse Reference Atlas (http://atlas.brain-map.org). Black arrows in right panels indicate cryptococcal cells. Scale bars: **A** = 1047 µm; **B**–**D** left panels = 200 μm; **B**–**D** right panels = 50 μm.

Uncontrolled cryptococcal infection, can lead to the formation of localized CNS lesions or cryptococcomas (Figure 2A), a biofilm-like collection of yeast cells entangled in capsular material and characterized by neuronal loss (Figure 2B). This cryptococcal lesion, often surrounded by microglia [59], is an innate immune response typically observed in infected individuals unable to fight against the overwhelming cryptococcal burden (Figure 2C). Instead of the microbicidal response, the biofilm-like mass is encased with lymphocytes, macrophages and multinuclear giant cells, resembling a chronic granulomatous reaction (Figure 2D) [60]. Interestingly, *C. neoformans* derived vesicles were found to be distributed inside and around the lesions, further bolstering the role of vesicles in its pathogenesis [61]. This mechanism may function as a method of adaptation to local tissue environments favoring *C. neoformans* dormancy, from which the yeast can become reactivated during an immunosuppressive episode in the host leading to an aggressive infection. The prognosis of patients with signs of a suspected cryptococcoma is dependent on the location of the lesion. For instance, there have been reported cases where patients, including an 11-year-old immunocompetent child, present with common signs and symptoms of CNS tumors [62,63,64]. Imaging studies revealed ring-enhancing mass lesions in these patients, and post-operative histopathologic studies with Grocott-Gomori methenamine silver and mucicarmine stains confirmed *C. neoformans* involvement [62].

A unique and under studied factor contributing to *C. neoformans* virulence is its ability to synthesize melanin *in vitro* [65,66] and *in vivo* [67] through a process where catecholamines [68], abundant in the CNS, are oxidized by copper-containing enzyme laccase [69]. The protective efficacy of melanin was demonstrated when deletion of both cryptococcal laccases, *lac1* and *lac2* [70] resulted in reduced survival of *C. neoformans* following ingestion by primary macrophages [71]. Hence, cryptococcomas are an ideal environment for melanin synthesis and may play a protective role against oxidative damage [72,73]. *C. neoformans* can use the neurotransmitters dopamine or norepinephrine as a substrate to undergo rapid melanization resulting in pigment deposition in the cell wall [65]. In addition, morphological changes of yeast cells during infection have not only been attributed to increased polysaccharide capsule size, but also thickening of the fungal cell wall [29]. For example, biofilms with melanized cryptococcal cells display increased resistance to antifungal drugs in vitro [19] and might protect the fungus against antifungal drug action and elimination by phagocytic cells, thereby exacerbating CNS disease.

## 2. Conclusions

The majority of microbiologic studies of infectious agents prior to the first decade of the 21st century were performed using planktonic microbial populations. Advances in confocal microscopy, biochemical, and big data acquisition techniques have made it possible to study microorganisms in single and inter-kingdom communities in the environment and humans as part of a functional microbiome. Currently, it is clear that microbial biofilms show variable phenotypic characteristics compared to those of their planktonic counterparts, including differences in metabolomics, proteomics, and community-based regulation of gene expression mediated by chemical communication or quorum sensing [74,75]. The importance of exploring *C. neoformans* pathogenesis is apparent when noting the microbe’s ability to form biofilms on medical devices and biofilm-like structures in tissue, particularly in the CNS. This organized architecture may augment communication processes in the fungal population, whereby secreted signaling molecules or quorum sensing may stimulate deliberate production of the fungus’ virulence factors enhancing tissue colonization and survival [14,76]. 

*C. neoformans* natural habitat, soil-containing pigeon droppings, is a source of constant environmental stressors and predation that may prepare the fungus to defy and evade challenging interactions with cells of the immune system [12]. However, there is limited information on how this encapsulated fungus has developed defensive mechanisms from environmental interactions to thrive in diverse climates and host species [14]. Growth conditions mimicking the external environment lead to biofilm formation, suggesting that this mode of growth may be an evolutionary adaptation as a protective niche [20,77]. Therefore, it is not surprising that interactions of *C. neoformans* with other microbes increase capsular synthesis and release, as well as biofilm formation [14]. In order to understand *C. neoformans* behavior inside of the host, future studies must focus on elucidating the regulation of molecular pathways of capsular production after polymicrobial interactions. Likewise, cryptococcal symbiotic interactions may shed light on the elusive molecules involved in cryptococcal quorum sensing and their impact in capsular production, biofilm formation, melanin deposition, and many other potential virulence factors such as synthesis of phospholipase, urease, and mannitol. The use of big data acquisition techniques such as proteomics, metabolomics, and transcriptomics will provide novel and interesting data to monitor phenotypic changes and production of metabolites during these interactions [48]. The molecular mechanisms responsible for such changes, from a fungal point of view, are of great relevance for understanding the evolution of microbial survival and population dynamics in the fungus’ ecological niche and within the human host. 

Microglia play a vital role in controlling brain tissue colonization by microbes. In spite of their significance, there is limited information on the interactions of these CNS resident cells and *C. neoformans* [59]. Studies on the interplay between microglial cells and *C. neoformans* biofilms are warranted since microglia’s inability to control cryptococcal proliferation and occupation may explain the pathogen’s predilection for the CNS. Considering the high incidence of cryptococcal meningoencephalitis cases, particularly in immunosuppressed individuals in Sub-Saharan Africa [1], it is unacceptable that current and comprehensive studies focusing on microglia and the impact of peripheral macrophages are severely lacking. Additionally, further investigations of the effect of *C. neoformans* and its virulence factors, particularly GXM, on neurons and astrocytes are imperative for a clearer picture of the CNS disease. 

Finally, application of novel methods such as combination of laser microdissection technology and nanoproteomic techniques may be helpful for isolation of biofilm-like structures and enhanced understanding of the cryptococcoma environment in order to develop antifungal strategies to eradicate *C. neoformans* from the CNS. Our efforts should focus on the integration of interdisciplinary fields including infectious diseases, immunology, neurosciences, structural and system biology, and drug discovery for the development of strategies to either prevent or eradicate CNS fungal colonization. Creating novel avenues to ascertain the molecular mechanisms of cryptococcal biofilm formation with concomitant development of novel anti-biofilm agents may be crucial to minimizing the devastating effects of this infection in individuals with impaired immunity.
